# Cell-free nucleic acid patterns in disease prediction and monitoring—hype or hope?

**DOI:** 10.1007/s13167-020-00226-x

**Published:** 2020-10-29

**Authors:** Adriana Torres Crigna, Marek Samec, Lenka Koklesova, Alena Liskova, Frank A. Giordano, Peter Kubatka, Olga Golubnitschaja

**Affiliations:** 1 Department of Radiation Oncology, University Hospital Bonn, Rheinische Friedrich-Wilhelms-Universität Bonn, Bonn, Germany; 2 Department of Obstetrics and Gynecology, Jessenius Faculty of Medicine, Comenius University in Bratislava, 03601 Martin, Slovakia; 3 Department of Medical Biology, Jessenius Faculty of Medicine, Comenius University in Bratislava, 03601 Martin, Slovakia; 4 Predictive, Preventive, Personalised (3P) Medicine, Department of Radiation Oncology, University Hospital Bonn, Rheinische Friedrich-Wilhelms-Universität Bonn, Bonn, Germany

**Keywords:** Predictive preventive personalised medicine (PPPM/3PM), Cell-free nucleic acids (CFNAs), miRNA, Biomarker panel, Methylation status, Physical activity, Apoptosis, Systemic hypoxic-ischemic lesion, Tumour development progression, Cancer, Stroke, cfDNA, ctDNA, Diet, Blood, Plasma, Serum, Saliva, Stress, Associated disease, Cardiovascular, Neurologic, Virus, COVID-19, Liquid biopsy, Therapy monitoring, Qualitative and quantitative analysis, Precancerous lesions, Mutations, Breast cancer, Colorectal cancer, Lung cancer, Prostate cancer, Diabetes, Metabolic disorder

## Abstract

Interest in the use of cell-free nucleic acids (CFNAs) as clinical non-invasive biomarker panels for prediction and prevention of multiple diseases has greatly increased over the last decade. Indeed, circulating CFNAs are attributable to many physiological and pathological processes such as imbalanced stress conditions, physical activities, extensive apoptosis of different origin, systemic hypoxic-ischemic events and tumour progression, amongst others. This article highlights the involvement of circulating CFNAs in local and systemic processes dealing with the question, whether specific patterns of CFNAs in blood, their detection, quantity and quality (such as their methylation status) might be instrumental to predict a disease development/progression and could be further utilised for accompanying diagnostics, targeted prevention, creation of individualised therapy algorithms, therapy monitoring and prognosis. Presented considerations conform with principles of 3P medicine and serve for improving individual outcomes and cost efficacy of medical services provided to the population.

## Liquid biopsy is instrumental for predictive diagnostics and targeted treatments

Liquid biopsy (LB) and individualised profiling of biomarker patterns presented in body fluids represent a revolutionary approach in the workframe of 3P medicine [1]. Current paper is dedicated to the liquid biopsy utilising specifically blood samples as the best explored source of information amongst other sorts of body fluids [2].

In the last years, cell-free nucleic acids (CFNAs) “signature” attracted a lot of attention for diagnostic and treatment purposes. Altered profiles of CFNAs have been detected under physiological conditions, e.g. by making sport, suboptimal conditions such as overtraining syndrome in physical exercises [3], acute and chronic pathological conditions including sepsis, stroke, trauma, myocardial infarction, autoimmune diseases and cancers [4]. To this end, certainly the area of oncological research is particularly advanced implementing ctDNA and miRNA detection and quantification for diagnostic and treatment purposes [5].

However, independently of the application area, the main goal remains the same, namely to look for pathology-specific patterns [6–8] as well as for patterns clearly indicating associated risks, for example, in vasospastic individuals who may be particularly predisposed to an increased stress sensitivity [9–11], neuro/degenerative pathologies [12, 13] and/or aggressive metastasing cancers [14, 15]. 

## Diagnostic and prognostic potential of cell-free nucleic acids’ signature in stress conditions and stress-related pathologies

Dysregulation at the level of CFNAs acts as a promising diagnostic biomarker panel for measuring imbalanced stress and for predicting stress-associated pathologies. According to the World Health Organisation (WHO), stress presents the epidemic of the third millennium [16]. Accumulated evidence suggests a tight association between chronic stress and psychiatric disorders [17–21]. Especially severe, prolonged and/or chronic stress of any origin such as exercise-induced oxidative stress [22] (see “Physical activity and exercise-induced oxidative stress” section), hormonal stress [23], emotional stress and psychological burden [24–27] as well as metabolic stress, e.g. in diabetes mellitus [28, 29] (see also below “Association between diabetes mellitus and carcinogenesis: diagnostic and therapeutic potential of cell-free nucleic acids” section) and hyperhomocysteinaemia [30, 31] amongst others, is associated with highly increased ROS production and insufficient repair capacity—both linked to oxidative damage of mitochondria and consequent mitochondrial dysfunction leading to the development of cardiovascular impairments [32–34], neuro/degenerative pathologies [34–37], impaired healing [34] and malignant cell transformation [34, 38–42]. Noteworthy, the pathomechanisms carry a systemic character [43] that is crucial for tracing corresponding alterations in a minimally invasive manner utilising blood samples and other body fluids [1].

An application of liquid biopsy is a promising approach to identify biomarker patterns specific for stress and stress-associated diseases. Prominent examples are summarised below.

Acquired data revealed lower expression of serum miR-183 and miR-212 in major depressive disorder (MDD) patients after antidepressant therapy [44]. Further, miR-16, miR-135a and miR-1202 were significantly reduced in serum of patients diagnosed with depression compared with healthy individuals [45]. Plasma miR-134 (associated with the regulation of synaptic plasticity and neurogenesis) was downregulated in a cohort of patients with MDD compared with healthy controls. Measurements of miR-134 patterns are also useful to distinguish between MDD, bipolar disorder and schizophrenia [46]. Further, an increased expression of miR-124-3p has been detected in serum of antidepressant-free MDD patients compared with healthy controls [47]. Another study revealed significantly higher levels of plasma miR-451a and lower levels of miR-320 in a group of depressed patients [48]. Another study detected significantly higher levels of serum miR-221-3p, miR-34a-5p and let-7d-3p in patients with MDD compared with controls [49]. Moreover, depressive symptoms were associated with the downregulation of plasma miR-144-5p considered as a useful biomarker for pathological processes associated with depression [50].

Posttraumatic stress disorders (PTSDs) as a consequence of acute traumatic stress demonstrate specific patterns of miR-142-5p, miR-19b, miR-1928, miR-223, miR-332, miR-324, miR-421-3p, miR-463 and miR-674. Anxiety and delayed fear are reflected in specific patterns of the panel comprising miR-142-5p, miR-1928, miR-223 and miR-19b [51] as detected, for example, in veterans suffering from PTSDs. To this end, miR-203a-3p derived from extracellular vesicles was upregulated, whilst miR-339-5p was downregulated in a cohort of PTSDs patients compared with controls [52]. Differentially expressed circulating miRNAs associated with PTSDs were detected in another study focused on stress-related disorders in the population of military veterans [53].

In a preclinical study, miR-24-2-5p, miR-27a-3p, miR-30e-5, miR-3590-3p, miR-532-5p and miR-362-3p patterns were decreased in rats with manifested vulnerability to chronic stress, whereas another panel comprising miR-28-3p, miR-139-5p, miR-326-3p and miR-99b-5p was downregulated in rats more resistant to stress—both compared with controls [54].

In the context of stress, cfDNA is an excellent biomarker candidate for clinical application considering circulating cell-free mitochondrial DNA (ccf-mtDNA). Correlation between serum ccf-mtDNA and psychological stress was demonstrated in the study focused on the cohort of healthy midlife adults. A brief psychological challenge in tested volunteers led to increased serum ccf-mtDNA, in contrast to circulating cell-free nuclear DNA [55]. Increased plasma concentrations of ccf-mtDNA have been demonstrated also for patients diagnosed with MDD and concluded as a biomarker associated with psychiatric disorders and useful for monitoring the pathology development and therapy response [56]. Table 1 summarises CFNAs associated with stress.

**Table 1:** CFNAs in psychological stress and stress-associated pathologies; explanatory note: ↑ increased levels, ↓ decreased levels

Biomarker	Liquid biopsy samples	Experimental design	Study results	Reference
miRNA	Serum	Patients (*n* = 33) with MDD diagnosed and treated with antidepressants	↓ miR-183; -212	[44]
miRNA	Serum	Patients (*n* = 39) with depression versus disease-free controls (*n* = 36)	↓ miR-16; -135a; and -1202	[45]
miRNA	Plasma	Patients with MDD (*n* = 100), bipolar disorder (*n* = 50), schizophrenia (*n* = 50) versus disease-free controls (*n* = 100)	↓ miR-134	[46]
miRNA	Serum	Patients (*n* = 18) with MDD versus disease-free controls (*n* = 17)	↑ miR-124-3p	[47]
miRNA	Plasma	Patients (*n* = 50) with depression versus disease-free controls (*n* = 41)	↑ miR-451a ↓ miR-320	[48]
miRNA	Serum	Patients (*n* = 32) with MDD versus disease-free controls (*n* = 21)	↑ miR-221-3p; -34a-5p; and let-7d-3p	[49]
miRNA	Plasma	Patients (*n* = 169) with depressive disorders versus disease-free controls (*n* = 52)	↓ miR-144-5p	[50]
miRNA	Serum	Sprague Dawley rat model of PTSDs	Dysregulation of miR-142-5p; -19b; -1928; -223-3p; -332; -324; -421-3p; -463; and -674	[51]
miRNA	Plasma	Military veterans with PTSDs (*n* = 10) and without PTSDs (*n* = 10)	↑ miR-203a-3p ↓ miR-339-5p	[52]
miRNA	Peripheral blood	Combat veterans (*n* = 24) with and without PTSDs	↑ miR-19a-3p; -101-3p; 20b-5p; -20a-5p ↓ miR-486-3p; -128-3p; -15b-3p; -125b-5p;	[53]
miRNA	Blood	Rat model of chronic social defeat	↓ miR-24-2-5p; -27a-3p; -30e-5; -3590-3p; -532-5p and -362-3p	[54]
ccf-mtDNA	Serum	Participants (*n* = 50) exposed to brief psychological challenge	↑ ccf-mtDNA	[55]
ccf-mtDNA	Plasma	Individuals (*n* = 50) with MDD versus disease-free controls (*n* = 55)	↑ ccf-mtDNA	[56]

## Physical activity and exercise-induced oxidative stress

Regular physical activity defined as movements of body mediated by skeletal muscles resulted in the energy expenditure usually measured in kilocalories [57] has been demonstrated as being crucial for physical and mental health benefits [58], prevention of various diseases including metabolic syndrome, obesity, insulin resistance, atherosclerosis, diabetes, neurodegenerative diseases and cancers [23–27]. Regular individually adapted exercise has an ability to inhibit ROS production, ameliorates the antioxidant capacity and improves mitochondria efficiency reducing oxidative stress and cellular damage [59]. Temporary increased levels of inflammation and cfDNA were observed in various acute exercises such as marathon, ultramarathon, resistance exercise, continuous, interval, and incremental treadmill running, and incremental rowing exercise [60–63]. However, during the period of physiologic recovery, the cfDNA levels usually come back to the baseline level [64]. In contrast, overtraining causes exercise-induced oxidative stress [22]. Consequently, the question is—how to distinguish between beneficial physical activity on one hand and damaging exercise-induced oxidative stress on the other hand, when providing recommendations at individual level? Circulating CFNAs might be helpful answering this question, since their patterns strongly depend on the intensity and duration of exercise being complementary to specific metabolic markers such as lactate and creatine kinase recognising muscle damage [3, 65]. To this end, the overtraining and induced inflammation are well reflected in C-reactive protein (CRP) levels as the marker of inflammation and highly increased concentration of plasma cfDNA in proportion to training load [66]. In addition, there is no any significant difference in circulating cfDNA between obese and normal-weight subjects [67]. Noteworthy, although remaining unchanged in its absolute quantity, the proportional input by the foetal cfDNA is reduced in mother’s blood by increased concentration of cfDNA linked to the exercise during and immediately after the physical activity. This proportion is normalised by 30 min after the exercise is finished [68].

The initiative called *Education Outside the Classroom* (EOtC) promoting physical activity against obesity in youth, has demonstrated increased level of cfDNA for both—sedentary behaviour and moderate-to-vigorous physical activity groups. Based on the results, the authors recommend light physical activity with the best potential to be supportive for health in examined children [69]. Further, diabetes predisposition can be diagnosed, e.g. in persons with sedentary lifestyle by applying miR-192 and miR-193b panel detected in the prediabetic stage but not in diabetic patients. Moreover, in glucose-intolerant mice and prediabetic individuals, regular exercises as a therapeutic strategy have normalised the miRNA patterns to the baseline level [70]. Furthermore, in healthy subjects, 74 circulating miRNAs associated with various heart diseases were evaluated at baseline, immediately after exercise and after 24 h. Only miR-103a-3p was reduced in both types of exercises: 10 km and marathon races. Furthermore, increased serum levels of miR-132-3p and miR-150-5p were detected forthwith after the 10-km race. On the contrary, decreased serum levels of miR-103a-3p, miR-590-5p and miR-139-5p were observed in the same type of exercise. Moreover, decreased levels of miR-103a-3p and miR-375-5p were observed immediately after marathon race and remained low also after 24 h. Further, several cardiac markers were upregulated and lasted for 24, 48 and/or 72 h after both exercises. Taken together, circulating miRNAs can be useful for patients with dysfunction symptoms after an acute attack of endurance physical activity [71]. Additionally, increased levels of circulating miRNAs including miR-126, miR-130b, miR-221 and miR-222 at baseline levels were detected in obese versus normal-weight subjects. These patterns but at higher levels were observed after acute aerobic exercise in obese subjects, even after controlling for VO_2max_ and insulin resistance (HOMA-IR) [72].

In summary, specific CFNAs patterns have been demonstrated in relationship to physical activity that allows to clearly differentiate between beneficial physical activity and exercise-induced oxidative stress and to provide accompanied diagnostics and individualised recommendations for healthy individuals and athletes, individuals in suboptimal health as well as for a variety of patients. Corresponding information is summarised in Table 2.

**Table 2:** Differences in CFNAs levels after acute or chronic exercises; explanatory note: ↑ increased levels, ↓ decreased levels

Biomarker	Liquid biopsy samples	Experimental design	Type of exercise	Study results	Reference
cfDNA	Plasma	17 Recreationally trained men (healthy volunteers); age, 21.56 (2.6) years; body weight, 77 (7.1) kg; body height, 1.77 (0.11) m; body fat, 12.2 (2.1) %	12-week resistance training regimen: 8 resistance multi-joint exercises selected to stress the entire musculature: bench press, squat, leg press, snatch, hang clean, dead lifts, barbell arm curlsand rowing	↑ Cell-free plasma DNA during t1, t2 and t3; ↑ CRP, ↑ creatine kinase; ↑ uric acid	[66]
cfDNA	Plasma	*n* = 14 (7 obese and 7 normal-weight) healthy male subjects in the age of 18–45	Treadmill—acute high-intensity interval exercise (30 min of total exercise, including a 5-min warm-up period of walking/jogging)	Both obese and normal-weight male: ↑ cfDNA, ↑ IL-8	[67]
cfDNA	Plasma	Nine pregnant women carrying male foetuses at gestational age 12(+0) weeks to 14(+6) weeks	Cycling	↓ Foetal cfDNA fraction, ↑ cfDNA of pregnant women	[68]
cfDNA	Saliva	EOtC programme: fifth-grade students *(n* = 37 with outdoor lessons), control group (*n* = 11 with indoor lessons) (fall/spring/summer)	Light physical activity and moderate-to-vigorous physical activity	Students with outdoor lessons: ↓ cortisol, ↑ cfDNA	[69]
Circulating miR-192 and miR-193b	Serum	*n* = 92 male individuals with different degrees of glucose tolerance; 6-week-old C57BL/6J male mice	Regular exercise (exercise programme: twice per week for 16 weeks)	Prediabetic humans and glucose-intolerant mice: ↑ miR-192, ↑ miR-193b;prediabetic humans and glucose-intolerant mice with regular exercise: ↓ miR-192, ↓ miR-193b	[70]
74 Circulating miRNAs	Serum	Healthy, highly trained middle-aged amateur subjects (*n* = 9)	10-km race (half-marathon) and marathon	10-km race: ↓ miR-103a-3p, ↑ miR-132-3p, ↑ miR-150-5p, ↓ miR-590-5p, ↓ miR-139-5p Marathon: ↓ miR-103a-3p, ↓ miR-103a-3p, ↓ miR-375-5p	[71]
Circulating miR-126, miR-130b, miR-221, miR-222	Plasma	*N* = 24 (12 normal-weight and 12 obese) subjects	30-min aerobic exercise (75% VO_2max_).	After acute aerobic exercise in obese subjects: ↑ miR-126, ↑ miR-130b, ↑ miR-221, ↑ miR-222	[72]

*EOtC =* education outside the classroom, *CRP = C*-reactive protein, *t1, t2, t3* = time poins*, IL-8* = interleukin 8

## Ischemic lesions and stroke

Stroke is one of the leading and preventable causes of sudden death and the most common cause of long-term disability worldwide [73, 74]. Ischemic stroke (IS) accounts for approximately 80–85% of stroke cases against haemorrhagic one [75, 76]. In short, IS is associated with a cascade of events including cerebral ischemia, obstructions in cerebral blood flow, generation of reactive oxygen species, inflammatory processes, neuronal damage and apoptosis leading to neurological dysfunction [77]. IS is a heterogeneous, multifactorial disease associated with an interaction between genetic and modifiable risk factors [74]. Besides evident genetic predisposition, dietary patterns and lifestyle-related stressors strongly contribute to the development of IS [78]. Current diagnostic approaches applied for IS are not rarely associated with some obstacles such as prolonged time of the imaging performance, poor sensitivity and / or data interpretation, particularly in case of asymptomatic clinical picture [78]. To this end, so-called young stroke—the rapidly increasing patient cohort below 50 years of age with unclear aetiology—is particularly challenging for healthcare globally [13] demanding innovative solutions in the framework of 3P medicine. Phenotyping and blood-based biomarkers are currently under extensive consideration for the risk assessment and predictive diagnosis of IS [13, 79]. To this end, the blood-brain barrier may prevent releasing brain-specific molecules into the bloodstream [80]. However, due to ischemia-related progressive cell death and consequent blood-brain barrier breakdown, the cfDNA release into the blood might accompany IS [77]. Moreover, due to chronic systemic effects, e.g. in vasospastic individuals predisposed to IS [13], a significant increase in the cfDNA blood concentration may happen days and weeks before the acute IS event.

Indeed, the cfDNA concentration correlates well with the severity at admission and with individual outcomes in IS patients [81] supporting meaningful measurements of plasma nuclear and mitochondrial cfDNA patterns [82] including specificity of the DNA fragmentation (300–400 bp range) profiling for diagnostic and prognostic purposes [77, 81–83].

miRNA panels provide complementary information in overall IS diagnostics: circulating exosomal miR-223 is significantly increased in acute IS against healthy controls, and its level correlates with stroke severity and individual outcomes [84]. In contrast, serum miR-221-3p and miR-382-5p patterns are downregulated in IS patients against healthy controls [85]. Moreover, the combination of miR-21-5p and miR-30a-5p was demonstrated as being of great utility to distinguish between hyper-acute, subacute and recovery phase of IS [80]. The miRNA panel comprising PC-3p-57,664, PC-5p-12,969, miR-122-5p and miR-211-5p demonstrates a correlation between upregulation in IS patients and post-mortem IS-brain specimens [86].

Table 3 summarises information on CFNAs in IS.

**Table 3:** CFNAs as a biomarker panel in ischemic stroke

Biomarker	Liquid biopsy samples	Experimental design	Study results	Reference
cfDNA	Plasma	Ischemic stroke patients (*n* = 26)	Correlation of cfDNA levels with severity of stroke at admission and poor outcome within 3 months	[81]
cfDNA	Plasma	Ischemic stroke patients (*n* = 54)	Higher cfDNA associated with severity at the time of admission and poor outcomes	[77]
cfDNA fragments	Plasma	Ischemic stroke patients (*n* = 48) versus healthy controls (*n* = 20)	High abundance of plasma cfDNA fragments (300–400 bp) in ischemic stroke patients versus healthy controls	[83]
Plasma nuclear and mitochondrial DNA	Plasma	Acute ischemic stroke patients (*n* = 50) versus at risk control subjects (*n* = 50)	Higher plasma nuclear and mitochondrial DNA in acute ischemic stroke patients versus subjects at risk Persistence of higher plasma nuclear DNA within 1 month after acute IS event Positive correlation between plasma nuclear DNA and clinical severity	[82]
Exosomal miR-223	Serum	Acute ischemic stroke patients within 72 h (*n* = 50) versus healthy controls (*n* = 33)	Exosomal miR-223 correlated with NIHSS scores Higher exosomal miR-223 associated with acute ischemic stroke occurrence Higher exosomal miR-223 in patients with poor outcomes versus patients with good outcomes	[84]
Exosomal miRNA-21-5p in combination with miRNA-30a-5p	Plasma	Ischemic stroke patients (*n* = 143)	Diagnosis of ischemic stroke Distinguishing between hyper-acute, subacute and recovery phases of ischemic stroke	[80]
miRNA-221-3p and miRNA-382-5p	Serum	Ischemic stroke patients (*n* = 78) versus healthy controls (*n* = 39)	Downregulated miRNA-221-3p and miRNA-382-5p in ischemic stroke patients versus controls	[85]
miRNAs panel (PC-3p-57664, PC-5p-12969, miR-122-5p, miR-211-5p)	Serum	Ischemic stroke patients (*n* = 34) versus healthy controls (*n* = 11)	Correlation between upregulation in ischemic stroke patients and post-mortem ischemic stroke-brain specimens	[86]

*NIHSS* National Institutes of Health Stroke Scale

## Precancerous lesions and early cancer detection

Liquid biopsy application to early cancer detection demands highly sensitive detection methodology in order to track circulating tumour DNA (ctDNA) amounts or diverse sub/cellular structures secreted by precancerous lesions and/or at initial stages of cancer. For instance, testing viral sequences related to tumours, such as human papillomavirus (HPV) or Herpesvirus family (Epstein-Barr virus (EBV), cytomegalovirus (CMV) [87]), along with ctDNA methylation analysis is instrumental for diagnosing HPV-derived precancerous lesions. HPV 16 and 18 strains have been described as related to high risk, potentially leading to cervical cancer [88]. A cervical precancerous state is characterised by changes in collar cells making them more susceptible to cervix cancer development within 10-year time span, if not treated in a timely manner [89]. A meta-analysis study showed that despite the existing heterogeneity amongst studies, HPV cDNA detection is a specific and relatively sensitive tool for cervical cancer diagnosis [89]. Another study revealed the presence of HPV in 98.4% of tumours, being HPV16 and 18 dependent for 89.4% of cervical cancer patients detected [87]. Likewise, RNA-seq database indicated the presence of HPV in the majority of cervical cancers [90]. In addition, EBV, CMV and Herpesvirus 6 (HHV6) were detected in 21 and 18% of rectal and colon cancers, respectively. EBV was found to be associated with 23% of stomach cancers. Herpesviruses are often detected in stomach, colon and rectum cancers. Some types of liver cancers have been linked with hepatitis B and C virus (HBV and HCV). HPV was present in a small amount of bladder cancers along with a subset of head and neck cancers [87].

Premalignant neoplastic lesions, in particular, adenomas have often been detected to have distinct miRNA expression patterns. In a study assessing miRNA expression profiles of CRC and adenomas miR18a was upregulated in adenoma patients versus healthy controls [91]. A further study revealed ratios amongst three circulating miRNA to allow discriminating between benign prostate adenoma (hyperplasia) and prostate cancer in a more specific manner than standardised prostate-specific antigen (PSA) levels [92]. Another study described the use of several non-invasive biomarkers concomitantly (PSA together with androgen receptor CAG analysis and promoter methylation analysis) increasing predictive power of the prostate cancer and allowing its discrimination from benign prostate hyperplasia in 70–80% of cases [93].

Furthermore, quantitative and qualitative cfDNA characterisation has been described as capable to detect certain cancer types [94, 95], although being challenging yet for cancer screening application [96], since cfDNA origin, specificity and release kinetics have still to be clarified [97–100]. Plasma levels of short and long fragmented DNA and total cfDNA in oral cancer and precancerous lesions were evaluated and quantified. Results demonstrated an increased cfDNA concentration and integrity of DNA in oral cancer compared with other cohorts, rendering it a tool for early oral cancer detection [101]. Further study evaluated somatic circulating mutations in patients with breast, lung, colorectal and ovarian cancers to assess cancer disease staging [102]. Data revealed overall significant increase of cfDNA in cancer patients’ plasma compared with healthy subjects. Thereby, breast cancer cohort demonstrated the lowest mutant allele fraction of ctDNA. Noteworthy, advanced disease stages III and IV correlated with higher amount of ctDNA compared with early disease stages I and II across all cancer cohorts [102].

## Chronic inflammation in carcinogenesis reflected in CFNAs signature

Chronic inflammation (together with infectious diseases related inflammation) is estimated to be responsible for approximately 25% of all cancer cases [103]. In the context of inflammatory milieu, epithelial and inflammatory cells secrete reactive oxygen and nitrogen species (ROS and RNS) causing DNA damage [104]. This DNA damage and mutagenic lesions, such as 8-oxo-7,8-dihydro-2′-deoxyguanosine (8-oxodG) and 8-nitroguanine, occur in organs undergoing inflammation, eventually driving carcinogenesis [105]. Furthermore, parasites, viruses (HPV, EBV and hepatitis virus) and bacteria are considered to be pathogenic agents carcinogenic to humans [105]. Inflammation may also be promoted by physical, chemical and immunological factors [103, 106]. Chronic inflammation induces tissue injury, due to genetic and epigenetic aberrations, nucleic acid, lipid and protein damage via to ROS/RNS production. This tissue damage may activate tissue regeneration resulting from stimulation of progenitor/stem cells. Thus, accumulation of mutations in stem cells by ROS/RNS may result in mutant stem cells or cancer stem cells leading to carcinogenesis [105]. Consequently, detection of specific cfDNA, miRNA and methylation patterns are considered of great clinical utility for early cancer detection [107].

Indeed, cfDNA is known to accumulate under chronic inflammation, due to decreased clearance [108]. cfDNA, nuclear and mitochondrial DNA are actively secreted and mediate many processes such as immunomodulation, tumour growth progression and inflammation [108]. For instance, prostate carcinogenesis and disease progression are known to be promoted by chronic inflammation [109–111]. Risk factors related to prostatic inflammation are frequently related to immunological, genomic and environmental factors such as physical trauma, urinary microbial infection, chemical irritation, unhealthy diet and abnormal body weight [112, 113]. Recruitment of leukocytes, namely macrophages, lymphocytes, granulocytes and monocytes to the prostate have been observed in the prostate cancer driven inflammation responses [114, 115]. In advanced stages of prostate cancers, elevated peripheral blood neutrophil-to-lymphocytes ratios were observed, portraying worse overall survival (OS) and reduced sensitivity to chemotherapy and to anti-androgens [116].

## Cell-free nucleic acids in cancer management

Although analysis of solid tumour tissues is a golden standard in oncology [117], tissue biopsies entail some risks for patients apart from being limited in identifying genetic heterogeneity or tracking neoplasm evolution alternations within a tumour [118]. Clinical and laboratory advances have broadened tumour-related diagnosis, prognosis and predictive measures. In fact, the use of cfDNA has marked a potential minimally-invasive alternative option for genomic diagnostics.

ctDNA was described to be the tumour-derived fraction of cell-free DNA secreted into the blood [119]. ctDNA patterns in blood are considered as being a potent analytical option alternative to solid tumour biopsies for cancer detection and monitoring, due to rapid, non-invasive and cost-effective biomarker identification [120]. Besides cfDNA/ctDNA, malignancy-related blood patterns include circulating miRNA, circulating tumour cells (CTCs) and exosomes [121, 122]. Notably, also saliva, cerebrospinal fluid (CSF), pleural fluid, urine and tears are prospective sources of tumour-originated material [123–127].

### ctDNA detection in a broad range of neoplasms

ctDNA, released by cancer cells, have been identified in a broad range of neoplasm types both in early and late cancer stages, displaying levels from < 1 to > 100,000 mutated DNA fragments per ml of plasma. In cancer patients, ctDNA fractions differ greatly, fluctuating from less than 0.1% to more than 90% of overall cfDNA. There is an obvious great variability amongst ctDNA detected in patients with differing cancer type; however, variations in ctDNA fraction amongst patients with analogous tumour type may be attributed to biological disparities, as well as varying cell death rates within tumour cells [128–130]. Even though there are studies describing a correlation amongst the amount of cfDNA, cancer status and disease progression [131, 132], others reveal cfDNA quantification to be insufficient as an independent diagnostic tool, lacking information about tumour development [133, 134]. cfDNA low circulation concentration together with its considerable proportion of fragmentation make it a challenging compound to analyse [135]. Furthermore, identification and evaluation of ctDNA within total cfDNA represent a great challenge in cancer detection [128–130]. Nevertheless, ctDNA bears the tumour specific molecular features capable of early cancer diagnosis and prediction as well as disease prognosis.

### ctDNA patterns advance diagnostic approach

ctDNA has been detected in cancer patients’ plasma prior to mainstream screening methods: mutation of KRAS2 and P53 in healthy subjects are described as related to an increased risk of developing bladder cancer within a period of 6 years [136]. Detection of plasma/serum DNA alterations at early tumour stages along with the current available markers renders ctDNA a useful diagnostic mean for early breast cancer [133]. Similarly, the quantification of DNA levels and microsatellite alterations in plasma DNA of lung cancer patients, suggested a correspondence with their clinical condition, serving additionally as non-invasive follow up assays [134]. In a metastatic breast cancer study a decrease in cfDNA integrity along with an increase in plasma cfDNA concentration has been described compared with healthy controls [137]. Another study analysed CRC patient samples in a quantitative and qualitative manner. Results revealed high plasma and serum cfDNA values at the time of surgery, which further increase in relapsed patients, confirming CRC and determining cancer status [94].

### ctDNA allows targeted cancer therapy

The ability of tracking therapy response is one of the most significant traits of liquid biopsies, particularly in therapies with resistance mechanisms. KRAS mutations in colorectal tumour (CRC) progression are associated with acquired resistance and reduced response to anti-epidermal growth factor receptor (EGFR) therapies [138]. In a study comparing KRAS and BRAF mutations in both metastatic CRC plasma cfDNA and tumour tissue, specificity and sensitivity of 100% for BRAF V600E mutation and 96% on KRAS point mutations have been demonstrated. Thus, this study reveals high potential for developing better personalised medical services [139]. Furthermore, KRAS mutations were analysed in CRC samples, aiming to determine prevalence of KRAS amplification and evaluate its overall sensitivity to EGFR therapies. In presence of this genetic lesion, a lack of responsiveness to anti-EGFR inhibitors was found [138]. Similarly, another independent study revealed KRAS mutations to be common determinants of acquired resistance in CRC cancer patients [140].

Multiregional and shotgun sequencing of circulating tumour plasma DNA has revealed the potential to assess molecular heterogeneity of overall disease [141]. In a study quantifying ctDNA from CRC patients, ctDNA determination could track tumour dynamics in patients subjected to chemotherapy or surgery, revealing a potential customisable genetic approach [128]. Similarly, ctDNA was detected in 97% of patients with metastatic breast cancer with somatic genomic modifications, and identified tumour dynamics greater than CA 15–3 or CTC [129]. Another study testing prostate cancer plasma samples determined the genomic scenario and disease progression through the analysis of ctDNA in a non-invasive manner [142].

### ctDNA in therapy monitoring and prognosis

Circulating tumour DNA patterns have been described as a non-invasive biomarker able to detect marginal disease residues after surgery or neoplastic therapies [143–146]. Detection of ctDNA at time of diagnosis in NSCLC patients together with residual ctDNA is associated with poor prognosis [147]. Furthermore, a study revealed the prognostic capacity of ctDNA in plasma from CRC patients to determine survival rates and increased patient recurrence [148]. Elevated levels of cfDNA and plasma mutant KRAS levels (pmKRAS) were described to be directly correlated, making plasma cfDNA to an alternative prognosis biomarker [149]. Similar data were published by Dawson et al. for the breast cancer patient cohort [129]. In an advanced non-small cell lung cancer (NSCLC) patient study, ctDNA appeared to be more sensitive to mutation detection than CTC [150]. An independent study also indicated ctDNA to be a potent prognostic biomarker [151].

Measuring plasma or serum ctDNA profiles to monitor cancer development is a promptly developing field with great clinical potential. Studies focused on ctDNA as a tool for cancer diagnostic, prediction and/or prognosis are summarised in Table 4. ctDNA analysis may reinforce its use as personalised treatments for cancer patients. Nevertheless, validating studies are essential to bring this tool into daily clinical practice.

**Table 4:** ctDNA as a biomarker for neoplastic detection, predictive diagnostics and prognosis

Procedure	Application	Reference
Diagnosis	Early detection	[94, 133, 134, 136, 137]
Prediction	Molecular heterogeneity	[141]
	Tumour dynamic assessment	[128, 129, 142]
	Determination of early treatment response	[138, 139]
	Acquired resistance	[138, 140]
Prognosis	Detection of marginal disease residues	[143–146]
	Survival and recurrence rate	[148]
	Tumour load determination	[149]

### miRNA panels in cancer management—prominent examples

Anomalous miRNA patterns have been correlated with pathogenicity of several human cancers [187]. Overexpression of miRNA in cancer prompts their action as tumour suppressors or oncogenes depending on the target [188]; some miRNA may act as both concomitantly. Tumour-related miRNA are more stable to processing than other molecules, making them optimal tumour biomarkers [127]. Studies utilising miRNA as non-invasive biomarkers for cancer detection are summarised in Table 5.

**Table 5:** Circulating miRNA panels for cancer detection, monitoring and prognosis

Cancer type	Biomarker	Liquid biopsy samples	Experimental design	Reference
CRC	miR-17-3p and miR-92 elevated in CRC patients	Plasma	Three phase study: (phase I) plasma and biopsies from *n* = 5 patients with CRC, *n* = 5 healthy subjects; (phase II) *n* = 25 CRC patients, *n* = 20 healthy subjects; (phase III) *n* = 90 CRC patients; *n* = 50 healthy subjects (control); *n* = 20 inflammatory bowel disease (IBD) patients and *n* = 20 gastric cancer (GC) patients were included to determine biomarker specificity	[152]
CRC	miR-15b, miR-18a, miR-19a, miR-19b, miR-29a and miR-335 upregulated in CRC patients, with respect to healthy subjects	Plasma	*n* = 123 newly diagnosed patients with sporadic colorectal neoplasia (*n* = 63 with CRC and *n* = 60 with AA) versus *n* = 73 healthy subjects (control)	[91]
CRC	miR-15b, miR-18a, miR-19a, miR-19b, miR-29a and miR-335 upregulated in CRC patients, with respect to healthy subjects	Plasma	*n* = 96 CRC patients, *n* = 101 diagnosed with AA versus *n* = 100 healthy subjects (control)	[153]
CRC	miR-18a and miR-200c	Plasma	*n* = 78 CRC patients versus *n* = 86 healthy subjects (control)	[154]
CRC	miR-17, miR-19a, miR-20a and miR-223	Serum	Two sample- set: 1-*n* = 30 CRC patients and 2-*n* = 100 CRC patients (control subjects n/d)	[155]
CRC	miR-17	Serum/plasma/faecal	Meta-analysis comprising 10 studies with a total *n* = 938 CRC patients and *n* = 638 healthy subjects (control).	[156]
CRC	miR-21 and miR-92a	Serum	*n* = 200 CRC patients, *n* = 50 advanced adenoma (AA) patients, *n* = 80 healthy subjects (control)	[157]
CRC	miR-29a and miR-92a	Plasma	*n* = 157 patients total (*n* = 120 CRC patients and *n* = 37 AA), *n* = 59 healthy subjects (control)	[158]
CRC	Upregulation of miR-21, miR-31 and miR-135b	Plasma	*n* = 66 CRC patients, *n* = 50 healthy subjects (control)	[159]
CRC	miR-19a-3p, miR-21-5p and miR-425-5p	Serum	*n* = 196 CRC patients, *n* = 138 healthy subjects (control)	[160]
CRC	miR-18a, miR-21, miR-22 and miR-25	Plasma	*n* = 67 CRC patients (control subjects n/d)	[161]
CRC	Exosomal miR-19a and miR-92a	Serum	*n* = 227 CRC patients, *n* = 28 healthy subjects (control)	[162]
CRC	Exosomal miR-21, miR-23a, miR-150, miR-223, miR-1229, miR-1246 and let-7a	Serum	*n* = 88 primary CRC patients, *n* = 11 healthy subjects (control)	[163]
Breast	miR-1, miR-92a, miR-133a and miR-133b (upregulated)	Serum	*n* = 132 breast cancer patients and *n* = 101 healthy subjects (control)	[164]
Breast	miR-182	Serum	*n* = 46 breast cancer patients and *n* = 58 healthy subjects (control)	[165]
Breast	miR-148b, miR-376c, miR-409-3p and miR-801	Plasma	*n* = 127 sporadic breast cancer cases and *n* = 80 healthy subjects (control)	[166]
Breast	miR-34a, miR-93 and miR-373	Serum	*n* = 120 patients with primary breast cancer (M0), *n* = 32 patients with overt metastasis (M1) and *n* = 40 healthy subjects (control)	[167]
Breast	miR-21 and miR-146a	Plasma	*n* = 14 breast cancer patients and *n* = 8 healthy subjects (control)	[168]
Breast	miR-10b, miR-21, miR-125b, miR-145, miR-155, miR-191 and miR-382	Serum	*n* = 61 breast cancer patients and *n* = 10 healthy subjects (control)	[169]
Breast	Increased miR-21 levels and decreased miR-92a levels	Serum	*n* = 100 serum samples of patients with primary breast cancer versus *n* = 20 healthy subjects (control)	[170]
Breast	Increased miR-21	Serum	*n* = 102 breast cancer patients of different stages versus *n* = 20 healthy subjects (control)	[171]
Breast	13 up-regulated miRNA and 46 downregulated miRNA (59 differentially expressed)	Whole blood	*n* = 48 early stage breast cancer patients versus *n* = 57 healthy subjects (control)	[172]
Breast	miR-16, miR-21 and miR-451 significantly increased and miR-145 significantly reduced in breast cancer patients	Plasma	Case-control cohort: *n* = 170 breast cancer patients versus *n* = 100 healthy subjects (control); validation test: *n* = 95 other types of cancers (blindly validated), *n* = 70 breast cancer patients, *n* = 50 healthy subjects (control)	[173]
Lung	miR-1254 and miR-574-5p	Serum	Discovery cohort: *n* = 11 patients with early-stage NSCLC versus *n* = 11 healthy subjects (control). Validation cohort: *n* = 22 patients versus *n* = 30 controls	[174]
Lung	miR-20a, miR-24, miR-25, miR-145, miR-152, miR-199a-5p, miR-221, miR-222, miR-223 and miR-320	Serum	*n* = 400 NSCLC serum patients versus *n* = 220 healthy subjects (control)	[175]
Lung	34 serum miRNA	Serum	Two sets: 1. training set: *n* = 25 adenocarcinoma (AC) patients versus *n* = 39 healthy subjects (control), 2. testing set: *n* = 22 AC, 12 squamous cell carcinomas (SCCs), *n* = 30 healthy subjects (control)	[176]
Lung	miR-21	Serum	*n* = 88 NSCLC patients versus *n* = 17 healthy subjects (control)	[177]
Lung	Increased miR-30d and miR-486 levels and decreased miR-1 and miR-499 levels	Serum	Total *n* = 303 patients: *n* = 30 patients with longer survival, *n* = 30 patients with shorter survival, *n* = 243 NSCLC patients in training set *n* = 120 and testing set *n* = 123	[178]
Lung	miR-155, miR-182 and miR-197	Plasma	*n* = 74 lung cancer patients (33 stages I–II, 41 stages III–IV) versus *n* = 68 healthy subjects (control)	[179]
Lung	Decreased miR-21 along with mir-15b, mir-17, mir-28-3p, mir-106a, mir-126, mir-142-3p, mir-148a, mir-197, mir-221 and mir-486-5p levels	Plasma	*n* = 40 plasma samples (*n* = 19 patients in the training set and *n* = 34 plasma samples from 22 patients from the validation set) and control were represented by 15 pools of 5–7 healthy subject plasma samples	[180]
Lung	miR-21, miR-126, miR-210 and miR-486-5p	Plasma	*n* = 58 NSCLC patients (30 Stage I–II, 28 Stage III–IV) versus *n* = 29 healthy subjects (control)	[181]
Lung	miR-21, miR-126, miR-155 and miR-223	Serum	*n* = 6919 patients versus *n* = 7064 healthy subjects (control)	[182]
Prostate	miR-18a	Peripheral blood	*n* = 24 prostate cancer (PC) patients, *n* = 24 benign prostatic hyperplasia (BPH) patients and * n* = 23 healthy subjects (control)	[183]
Prostate	miR-141	Serum	n/d	[184]
Prostate	miR-182-5p and miR-375-3p	Plasma	*n* = 252 prostate cancer patients versus *n* = 52 healthy subjects (control)	[185]
Prostate	miR-372	Serum	*n* = 20 serum samples from prostate cancer patients versus *n* = 20 healthy subjects (control)	[186]

#### Colorectal cancer

Ng et al. described the significant overexpression of miR-17-3p and miR-92 in plasma from CRC patients versus control subjects. miR-92 marker specifically differentiates CRC from gastric cancer markers, making it to more sensitive CRC marker [152]. Similarly, a meta-analysis observed an increase in miR-17 in plasma/serum/faecal levels of CRC patients, with 68% specificity [156]. miR-92a together with miR-21 was highly increased in serum samples, possessing prognostic value in CRC patients [156]. Further, results from the analysis of CRC patient plasma revealed significant upregulation of a miRNA panel (miR-15b, miR-18a, miR-19a, miR-19b, miR-29a and miR-335) depicting different miRNA expression patterns amongst CRC patients and healthy subjects [91]. Further experiments validated these results, with 91% sensitivity and 90% specificity for CRC and advanced adenoma (AA) detection and prognosis [153]. Plasma miR-18 [154] and serum miR-19a [155, 160] have also been described to be significantly increased in CRC patients in comparison with healthy controls. miR-29a has also shown an important role as a potential biomarker for CRC detection. Furthermore, miR-29a combined with miR-92a are capable to distinguish advanced CRC from healthy subjects with 83% sensitivity and 84.7% specificity [158].

miR-21 has been extensively reported in multiple cancers as promoting proliferation and tumour growth, being one of the most relevant diagnostic miRNA oncogenes in tumour onset [189]. A study testing 380 miRNA described 19 dysregulated miRNA in CRC patient plasma samples. miR-21 upregulation discriminated CRC patients from healthy subjects with a sensitivity and specificity of 90% [159]. Furthermore, two independent studies revealed upregulation of miR-21 levels in CRC patients compared with controls even years prior to the clinical manifestation of the disease [160, 161].

Exosomal miRNAs, although still insufficiently investigated, are increasingly applied as biomarkers for cancer detection featuring high specificity. For instance, increased serum levels of exosomal miR-19a and miR-92a in CRC patients against controls have been detected [162]. Similarly, upregulation of serum exosomal miR-21 and miR-23 amongst others, was described in CRC patients [163].

#### Breast cancer

In breast tumour studies, many differentially expressed miRNA have been detected in breast cancer patients compared with healthy women. miR-1, miR-92a, miR-133a and miR-133b have been described as some of the most prevalent upregulated biomarkers in breast cancer samples [164]. Another study revealed miR-182 serum levels to be increased in patients with breast cancer versus controls. Furthermore, serum miR-182 levels were significantly lower in oestrogen receptor (ER)- and progesterone receptor (PR)-positive breast cancer patients than those in ER- and PR-negative patients, demonstrating their clinical utility for breast cancer diagnosis [165]. Additionally, 4 upregulated plasma miRNA (miR-148b, miR-376c, miR-409-3p and miR-801) managed to discriminate breast cancer patients from controls [166]. miR-34a, miR-93 and miR-373 serum levels were distinguishable between M0 breast cancer patients on one hand and healthy subjects on the other hand, whilst miR-17 and miR-155 differentiated M0 from M1 patients [167]. Further studies described miR-21 and miR-146a as increased in plasma levels, therefore, distinguishing breast cancer patients from healthy controls [168]. Similarly, another study revealed miR-21 increased serum levels, which together with miR-10b, miR-125b, miR-145, miR-155 miR-191 and miR-382 are indicative for breast cancer occurrence [169]. Moreover, two independent studies have described miR-21 increase to be of importance to discriminate breast cancer patients from healthy women [170, 171]. Increased miR-21 concentrations corresponded with visceral metastasis [171]. miR-92 decreased levels along with elevated miR-21 were positively associated with lymph node detection and tumour size [170]. A microarray panel study analysing 1100 miRNAs found 59 differentially expressed miRNA in whole blood from early stage breast cancer patients against healthy individuals, from which 13 were up-regulated and 46 were downregulated [172]. Looking for differences specific for breast cancer, 8 up-regulated and one downregulated plasma miRNA were discovered: miR-16, miR-21 and miR-451 were significantly increased and miR-145 significantly reduced in breast cancer patients [173].

#### Lung cancer

To date, lung carcinogenesis molecular signature has been mainly monitored through mRNA systematic analysis along with detection of protein expression levels [190]. However, miRNA expression pattern analysis may portray novel diagnostic and prognostic tools for predictive and early lung cancer detection [191]. Indeed, a study assessing miRNA expression in early-stage NSCLC serum samples revealed significantly increased miR-1254 and miR-574-5p levels, allowing for discrimination of NSCLC patients from controls with a 77% and 82% specificity and sensitivity, respectively, and in the validation cohort with a 71% specificity and 73% sensitivity [174]. Furthermore, 10 serum miRNA (miR-20a, miR-24, miR-25, miR-145, miR-152, miR-199a-5p, miR-221, miR-222, miR-223 and miR-320) were detected to be differentially expressed in NSCLC serum patient samples compared with controls. This specific miRNA profiling was able to detect NSLCL 33 months prior to the clinical manifestation of the disease [175]. 34-miRNA signature model was created to detect early-stage NSCLC within a population of high-risk asymptomatic subjects with an 80% accuracy [176]. In another study, miR-21 increased levels positively correlated with lymph node and tumour-node metastases in NSCLC patients; shorter 3-year overall survival compared with patients with low levels of miR-21 expression was demonstrated [177]. Similarly, miR-21 as well as miR-126, miR-210 and miR-486-5p were detected as a potential NSCLC diagnostic panel, portraying 86.2% of sensitivity and 96.6% specificity [181]. Contrarily, another study found that miR-21 along with miR-15b, miR-17, miR-28-3p, miR-106a, miR-126, miR-142-3p, miR-148a, miR-197, miR-221 and miR-486-5p were decreased in poor prognosis lung cancer cases [180]. NSCLC serum patient study described increased levels of miR-30d and miR-486 together with decreased levels of miR-1 and miR-499 as correlated positively with poor NSCLC prognosis [178]. Plasma miRNA analysis revealed miR-155, miR-182 and miR-197 levels to be considerably higher in lung cancer patients than in controls with a sensitivity of 81.33% and a specificity of 86.76%. Higher pattern values were detected in patients with metastasis than in those without [179]. Furthermore, miR-21, miR-126, miR-155 and miR-223 (84% specificity and 83% sensitivity) have arisen as a potential biomarker signature for lung cancer detection [182]. Altogether, these studies suggest that corresponding miRNA panels (but individual miRNAs) have a predictive power for lung cancer detection.

#### Prostate cancer

Prostate cancer (PC) diagnosis, monitoring and prognosis are widely based on the androgen-regulated genes and prostate-specific antigen (PSA) [192]. In recent years, miRNA have been described to impact cancer features by either promoting (oncogenic miRNA) or suppressing (suppressive miRNA) tumour development and disease progression [193]. PC often presents with a deregulation of miRNA that may operate as oncogenes or tumour suppressors [194]. Indeed, increased miR-141 levels were shown in PC serum samples [184]. Increased expression of miR-18a was strongly correlated with promotion of PC, acting as an oncogenic miRNA allowing discrimination between PC and benign prostatic hyperplasia (BPH) [183]. Moreover, miR 182-5p and miR-375-3p blood levels were detected as non-invasive screening signature and potential prognostic biomarker for PC development [185].

There is an accumulated evidence of numerous miRNA tested in prostate cancer tissue samples acting as tumour suppressors [195, 196]. ERG is able to bind to miR-200b/a/429 assisting transcription in PC cells in tumour tissues [196]. Moreover, miR-135a-1 was described to act as a potential tumour suppressor in metastatic PC by aiming at EGFR [195].

Another study revealed serum circulating miR-372 involvement in the progression of human PC by aiming p65-mediated NF-κB/MMP-9/PSA signalling pathway. Thus, targeting miR-372/p65 interplay or interceding in miR-372 expression may present a valuable tool for diagnosis and treatment of PC patients [186]. However, studies addressing miRNA panels PC specificity for example against prostate inflammation are needed.

## Association between diabetes mellitus and carcinogenesis: diagnostic and therapeutic potential of cell-free nucleic acids

Diabetes mellitus gathers several metabolic diseases characterised by a chronic state of hyperglycemia. It can result in a deficiency in secretion of insulin, lack of insulin effect or both simultaneously. Different types of diabetes exist, namely type 1, type 2 and gestational diabetes, that differ in genetics and aetiology [197]. Type 1 diabetes (T1D) is an autoimmune disorder characterised by hyperglycemia and β cell destruction [198], whereas type 2 diabetes (T2D) is considered a metabolic syndrome.

### Diabetes and carcinogenesis

Published epidemiological evidences portray a correlation between diabetes and cancer risk [199]. There are several potential risk factors common to both diseases, such as age, gender, diet, physical activity and obesity, amongst others [200]. Diabetic patients present increased blood glucose levels, along with advanced glycation end-products (AGE) that eventually leads to higher levels of DNA damage [201]. Studies have described AGE capability to cause DNA strand breaks in colon and liver cells, as well as in murine podocytes. Metabolic stress, mitochondrial impairments and insufficient DNA repair increase risk of all-site carcinogenesis and cancer progression in diabetic patients [38, 201].

For example, correspondence between diabetes and CRC has been described in numerous studies [202]. In fact, a study revealed a 5-year decreased overall CRC, colon and rectal cancer survival (18, 19 and 16%, respectively) in patients with diabetes [203]. Another study showed an increased risk in diabetic women of developing CRC than men [204]. In women a direct risk by diabetes for breast cancer development has been described. A meta-analysis showed an increased cancer risk in diabetic women versus non-diabetic individuals [205]. A potential link between diabetes and breast cancer is promoted by oestrogen levels [206, 207].

In a lung cancer study contrasting lung cancer patients with and without diabetic history, diabetes was not a detrimental factor for lung cancer survival [208]. Prostate cancer and diabetes studies have resulted in dissimilar outcomes. For one, a meta-analysis study revealed diabetic men to have decreased risk of developing prostate cancer [209]. Another study described an increase in 29% in prostate cancer-related mortality in diabetic patients compared with non-diabetic subjects [210]. Obviously, a detailed patient stratification by individualised patient profiling is essential to bring more consensus in the data interpretation that allows for a disease prediction and of high quality personalised services to the patient [9].

Anti-diabetic drugs are known to decrease diabetes pathophysiological factors (high blood glucose and AGE), however, drugs such as metformin may also reduce risk of cancer in diabetic patients. In fact, studies have postulated anti-oxidant properties of anti-diabetic drugs and renin-angiotensin system inhibitors to potentially reduce cancer risk [211, 212].

### Diagnostic and therapeutic potential of cell-free nucleic acids in diabetes

Determining differentially expressed miRNA or differentially methylated β cell derived DNA might better identify T1D cohorts, as miRNA are known to be imperative in T1D pathogenesis and regulating β cell function [213]. The use of proinsulin/C-peptide (PI/C) ratios may support identification of β cell destruction in subjects prior to the development of T1D, serving as a non-invasive marker of β cell malfunction [214].

miRNA-375 has been reported as being one of the most abundantly expressed miRNA in β cells. In fact, mice lacking miR-375 appeared to have decreased β cell mass and increased glucagon secretion, resulting in a hyperglycemic state [215]. A similar study portrayed an overexpression of miR-375 in primary mouse islets [216]. Consequently, miR-375 was tested as a potential biomarker for diabetes. In fact, increased miR-375 was detected in mice prior to hyperglycemia onset [217]. miRNA-375 plasma levels were elevated in patients at 7 days post islet transplantation [218]. Serum miRNA sequencing analysis has identified miR-52, miR-24, miR-25, miR-26a, miR-27a, miR-27b miR-29a, miR-30a-5p, miR-148a, miR-181a, miR-200a and miR-210 as differentially expressed in T1D patients [219]. Further studies have tested miRNA patterns in immune cells from T1D patients, revealing an increased expression of miR-326 in lymphocytes from T1D subjects [220]. Another study determined decreased expression of miR-146 in PBMC from T1D patients against non-diabetic controls [221].

Evaluation of increased unmethylated insulin DNA in circulation is a key to detect evolution of T1D resulting from β-cell death [222]. Two independent studies revealed higher unmethylated to methylated insulin DNA ratios versus non-diabetic controls [223] and higher circulating levels of both methylated and unmethylated insulin DNA in early onset T1D patients [224]. Similarly, plasma cell-free DNA levels from new onset T1D and allogeneic islet transplantation subjects were higher than in controls [225, 226].

Furthermore, T2D patient’s serum was tested for specific miRNA profiles: T2D were compared with obese patients and healthy controls. Combined miR-138 and miR-503 patterns enabled to discriminate between diabetic and obese diabetic patients. Further, using miR-15b in combination with miR-138 and miR-376a may help to distinguish between T2D and obesity. This evidence makes serum miRNA profiling to a potential T2D predictive tool [227]. Furthermore, a study which investigated plasma miRNA profiles in T2D patients revealed diminishing plasma levels of 10 miRNA and a slight increase of miR-28-3p. In fact, analysis of miR-15a, miR-28-3p, miR-126, miR-223 and miR-320 represent a suitable T2DM signature array [228].

## Pathology-specific versus common cell-free nucleic acid patterns

Imbalanced stress- and ischemia-related disorders, diabetes and cancer share several risk factors such as toxic environment, suboptimal life-style and dietary habits, specific phenotypes, vasospasm, accelerated ageing and abnormal body weight (both underweight and obesity), amongst others [9, 10, 13, 113, 229–232].

To this end, diabetes mellitus has been demonstrated as a prominent example of cancer risk factor [200]. Unfortunately, in many cases, studies do assess potential biomarkers out of context of collateral pathologies and potentially related health conditions that has been strongly criticised in the literature [233]. Those deficits should be compensated via well designed further studies, on one hand to indicate common origin and molecular pathways involved in several and collateral pathologies [234]. On the other hand, pathology-specific patterns are of great value for predictive diagnostic purposes, targeted prevention and cost-effective personalisation of medical services [6, 235].

Table 6 provides examples for pathology-specific and Table 7 for common CFNA panels in health conditions and disorders which the current paper has referred to.

**Table 6:** Pathology-specific CFNA panels

Marker	Disease	Reference
miR-20a-5p	Stress	[53]
miR-16	Stress	[45]
miR-30e-5	Stress	[54]
miR-221-3p	Stress	[49]
miR-34-5p	Stress	[49]
miR-135a	Stress	[45]
miR-142-5p	Stress	[51]
miR-223-3p	Stress	[51]
miR-451a	Stress	[48]
miR-320	Stress	[48]
let-7d-3p	Stress	[49]
miR-124-3p	Stress	[47]
miR-125b-5p	Stress	[53]
miR-128-3p	Stress	[53]
miR-101-3p	Stress	[53]
miR-19a-3p	Stress	[53]
miR-19b	Stress	[51]
miR-20b-5p	Stress	[53]
miR-20a-5p	Stress	[53]
miR-15b-3p	Stress	[53]
miR-134	Stress	[46]
miR-144-5p	Stress	[50]
miR-183	Stress	[44]
miR-203a-3p	Stress	[52]
miR-212	Stress	[44]
miR-27a-3p	Stress	[54]
miR-324	Stress	[51]
miR-332	Stress	[51]
miR-339-5p	Stress	[52]
miR-3590-3p	Stress	[54]
miR-362-3p	Stress	[54]
miR-421-3p	Stress	[51]
miR-463	Stress	[51]
miR-486-3p	Stress	[53]
miR-532-5p	Stress	[54]
miR-674	Stress	[51]
miR-1202	Stress	[45]
miR-1928	Stress	[51]
miR-24-2-5p	Stress	[54]
miR-132-3p	Physical activity	[71]
miR-150-5p	Physical activity	[71]
miR-375-5p	Physical activity	[71]
C-reactive protein (CRP)	Physical activity	[66]
miR-103a-3p	Physical activity	[71]
miR-130b	Physical activity	[72]
miR-192	Physical activity	[70]
miR-193b	Physical activity	[70]
miR-590-3p	Physical activity	[71]
miR-382-5p	Ischemic stroke	[85]
miR-122-5p	Ischemic stroke	[86]
miR-211-5p	Ischemic stroke	[86]
PC-3p-57,664	Ischemic stroke	[86]
PC-5p-12,969	Ischemic stroke	[86]
miR-19a	CRC cancer	[91, 153, 155, 162, 203]
miR-135b	CRC cancer	[159]
miR-150	CRC cancer	[163]
miR-200c	CRC cancer	[154]
miR-335	CRC cancer	[91, 153]
miR-34a	Breast cancer	[167]
miR-382	Breast cancer	[169]
miR-146a	Breast cancer	[168]
miR-451	Breast cancer	[173]
miR-30d	Lung cancer	[178]
miR-142-3p	Lung cancer	[180]
miR-197	Lung cancer	[179, 180]
miR-486-5p	Lung cancer	[178, 180, 181]
miR-135a-1	Prostate cancer	[236]
miR-141	Prostate cancer	[184]
miR-182-5p	Prostate cancer	[185]
miR-372	Prostate cancer	[186]
miR-375-3p	Prostate cancer	[185]
Proinsulin/C-peptide (PI/C)	Diabetes	[214]
miR-15a	Diabetes	[228]
miR-26a	Diabetes	[219]
miR-27a	Diabetes	[219]
miR-27b	Diabetes	[219]
miR-52	Diabetes	[219]
miR-138	Diabetes	[227]
miR-146	Diabetes	[221]
miR-181a	Diabetes	[219]
miR-200a	Diabetes	[219]
miR-326	Diabetes	[220]
miR-375	Diabetes	[215–217]
miR-376a	Diabetes	[227]

**Table 7:** Common CFNA panels

Marker	Disease	Reference
miR-15b	CRC cancer-lung cancer-diabetes	[91, 180, 227]
miR-16	Stress-breast cancer	[44, 45, 173]
miR-17	CRC cancer-lung cancer	[155, 156, 180]
miR-18a	CRC cancer-prostate cancer	[91, 153, 154, 183]
miR-19a-3p	Stress-CRC cancer	[53, 160]
miR-19b	Stress-CRC cancer	[51, 91, 153]
miR-20a	CRC cancer-lung cancer	[155, 175]
miR-21	CRC cancer-breast cancer-lung cancer	[157, 159, 160, 163, 168–171, 173, 177, 180–182]
miR-21-5p	Ischemic stroke-CRC cancer	[80, 160]
miR-24	Lung cancer-diabetes	[175, 219]
miR-25	Lung cancer-diabetes	[175, 219]
miR-28-3p	Stress-lung cancer-diabetes	[54, 180, 228]
miR-29a	CRC cancer-diabetes	[91, 153, 158, 219]
miR-30a-5p	Ischemic stroke-diabetes	[80, 219]
miR-92a	CRC cancer-breast cancer	[158, 162, 164, 170]
miR-126	Physical activity-lung cancer-diabetes	[72, 180–182, 228]
miR-139-5p	Stress-physical activity	[54, 71]
miR-145	Breast cancer-lung cancer	[169, 173, 175]
miR-148a	Lung cancer-diabetes	[180, 219]
miR-155	Breast cancer-lung cancer	[169, 179, 182]
miR-182	Stress-breast cancer-lung cancer	[44, 165, 179]
miR-210	Lung cancer-diabetes	[181, 219]
miR-221	Physical activity-lung cancer	[72, 175, 180]
miR-221-3p	Stress-ischemic stroke	[49, 85]
miR-222	Physical activity-lung cancer	[72, 175]
miR-223	Stress-ischemic stroke-CRC cancer-lung cancer-diabetes	[51, 84, 155, 163, 175, 182, 228]
miR-320	Stress-lung cancer-diabetes	[48, 175, 228]

### Pathology-specific CFNA panels 

Predominant CFNAs signatures for stress are miR-3590-3p, miR-362-3p, miR-421-3p [51, 54]. Several experimental and clinical studies identified dysregulation in others stress associated miRNA panels: miR-183; -212; -16; 135a; -1202; -134; -124-3p; -451a; -320; -221-3p; -34a-5p; let-7d-3p; -144-5p; -142-5p; -19b; -1928; -223-3p; -332; -324; -463; and -674; -203a-3p; -339-5p; -19a-3p; -101-3p; 20b-5p; -20a-5p; -486-3p; -128-3p; -15b-3p; -125b-5p; -24-2-5p; -27a-3p; -30e-5; -532-5p [44–54]. Some studies analysed cfDNA [66–69] as well as circulating miRNA panels (miR-192; -193b; -126; -130b; -221; -222) [70–72] after acute and chronic exercise. Last but not least circulating miRNA (miR-223; -21-5p; -30a-5p; -221-3p; -382-5p; -122-5p; -211-5p; PC-3p-57,664; PC-5p-12,969) [80, 84–86], cfDNA and mtDNA could act as promising diagnostic and prognostic biomarkers of ischemic stroke [77, 81–83].

 The miR-19a dysregulation has been described in numerous studies related to CRC cancer patients compared with controls [91, 153, 155, 160, 162]. Moreover, miR-335 was also detected in CRC tumours by several different groups [91, 153]. Breast cancer specific biomarkers detected are miR-34a, miR-382, miR-146a and miR-451 [167–169, 173]. Patterns of miR-197 [179, 180] and miR-486-5p [178, 180, 181] have been analysed as pathology-specific biomarkers in lung cancer patients. Moreover, prostate cancer specific biomarkers are miR-135a-1, miR-141, and miR-372, amongst others [184, 186, 195].

One of the most prominent diabetes mellitus biomarker is miR-375 [215–217] along with further biomarkers described as specific for diabetes detection such as the panel of miR-138, miR-181a, miR-326 and miR-376a, amongst others [219, 220, 227]. Table 6 summarises pathology-specific CFNA panels.

### Common CFNA panels 

Comprehensive analysis demonstrated common CFNA panels amongst different diseases. For instance, miR-21 has been described to be present in CRC, breast and lung cancers. miR-21-5p and miR-223 are related to both ischemic stroke and CRC cancer. Furthermore, miR-223 has been described for lung cancer, ischemic stroke, diabetes and stress-related diseases. Lung cancer shares several miRNA markers with stress-related disorders (miR-28-3p, miR-182, miR-223 and miR-320) and physical activity (miR-126, miR-221 and miR-222). miR-16 and miR-182 are present in both breast cancer and stress-related diseases. Moreover, miR-15b, miR-24, miR-25, miR-28-3p, miR-126, miR-148a and miR-320 were present in lung cancer and diabetic patients. Dysregulation of miR-21, miR-145 and miR-155 have been found in both breast and lung cancer patients. Whereas, miR-15b, miR-17and miR-20a are common markers in CRC and lung cancers.

Table 7 summarises common CFNA panels. Further studies addressing interrelations amongst human disorders and shared CFNAs signature are essential.

## What is known about CFNAs signature utility in COVID-19 management?

Many research teams around the world are intensively working on prediction of the COVID-19 epidemics, protective measures to populations, therapeutic and vaccination issues. It has been clearly demonstrated that lack of specific diagnostic laboratory tools may lead to incorrect political decisions causing either unnecessary overprotection of the population that is risky for a long-term economic recession, or underprotection of the population leading to a post-containment pandemic rebound [237, 238].

Blood parameters are highly indicative for the patient stratification, disease cause and individual outcomes [239]. Patients demonstrating severe course of COVID-19-related disease suffer from cytokine storms and multiorgan failure [240]; however, the underlying mechanisms still remain uncertain. Available information demonstrates that profuse innate immune responses aggravate individual outcomes [241]. Viral infections have been described to prompt cellular necrosis, which amplifies anti-viral immune responses releasing damage associated molecular patterns (DAMPs) [242]. Severely affected cells and tissues intrinsically secrete CFNAs such as mitochondrial DNA (MT-DNA) [243]. It has been demonstrated that COVID-19 patients with increased levels of MT-DNA are at elevated death risk, necessity of ICU care and intubation. Consequently, cell-free MT-DNA is a potential biomarker for individualised survival status prediction [243].

## PPPM-related conclusions

LB and individualised profiling of biomarker patterns presented in body fluids represent a revolutionary approach in the work-frame of 3P medicine. In the last years, CFNAs signature attracted a lot of attention for diagnostic and treatment purposes. Altered profiles of CFNAs have been detected under both physiological and pathological conditions. Although oncological research is particularly advanced implementing CFNAs for diagnostic and treatment purposes, independently of the application area, the main goal remains the same, namely to look for pathology-specific biomarker patterns as well as for patterns clearly indicating associated risks, for example, in vasospastic individuals who are a prominent example of patients predisposed to an increased stress sensitivity, neuro/degenerative disorders and/or aggressive metastasing cancers as discussed above. This article highlights the involvement of CFNAs in local and systemic processes dealing with the question, whether specific patterns of CFNAs in blood, their detection, quantity and quality (such as methylation status) might be instrumental to predict a disease development/progression and could be further utilised for accompanying diagnostics, targeted prevention, creation of individualised therapy algorithms, therapy monitoring and prognosis. Presented considerations conform with principles of 3P medicine [244] and can be implemented for improving individual outcomes and cost-efficacy of medical services provided to the population.

## List of abbreviations

8-oxodG: 8-Oxo-7 8-dihydro-2′-deoxyguanosine
AA: Advanced adenoma
AGE: Advanced glycation end-products
BPH: Benign prostatic hyperplasia
ccf-mtDNA: Circulating cell-free mitochondrial DNA
cfDNA: Cell-free DNA
CFNA: Cell-free nucleic acid(s)
CMV: Cytomegalovirus
CRC: Colorectal tumours
CRP: C-reactive protein
CSF: Cerebrospinal fluid
CTC: Circulating tumour cells
ctDNA: Circulating tumour DNA
DAMPs: Damage-associated molecular patterns
EBV: Epstein-Barr virus
EGFR: Epidermal growth factor receptor
EOtC: Education Outside the Classroom
ER: Oestrogen receptor
HBV: Hepatitis B
HCV: Hepatitis C
HHV6: Herpesvirus 6
HPV: Human papillomavirus
IS: Ischemic stroke
LB: Liquid biopsy
MDD: Major depressive disorder
MT-DNA: Mitochondrial DNA
NSCLC: Non-small cell lung cancer
OS: Overall survival
PC PI/C: Prostate cancer proinsulin/C-peptide
pmKRAS: Plasma mutant KRAS
PPPM / 3 PM: Predictive preventive personalised medicine
PR: Progesterone receptor
PSA: Prostate-specific antigen
PTSD: Posttraumatic stress disorders
RNS: Reactive nitrogen species
ROS: Reactive oxygen species
T1D: Type 1 diabetes
T2D: Type 2 diabetes
WHO: World Health Organisation
